# Intra-Articular Route for the System of Molecules 14G1862 from *Centella asiatica*: Pain Relieving and Protective Effects in a Rat Model of Osteoarthritis

**DOI:** 10.3390/nu12061618

**Published:** 2020-05-31

**Authors:** Laura Micheli, Lorenzo Di Cesare Mannelli, Luisa Mattoli, Sara Tamimi, Enrico Flamini, Stefano Garetto, Jacopo Lucci, Emiliano Giovagnoni, Lorenzo Cinci, Mario D’Ambrosio, Cristina Luceri, Carla Ghelardini

**Affiliations:** 1 Department of Neuroscience, Psychology, Drug Research and Child Health-NEUROFARBA-Pharmacology and Toxicology Section, University of Florence, Viale Gaetano Pieraccini 6, 50139 Florence, Italy; laura.micheli@unifi.it (L.M.); lorenzo.cinci@unifi.it (L.C.); mario.dambrosio@unifi.it (M.D.); cristina.luceri@unifi.it (C.L.); carla.ghelardini@unifi.it (C.G.); 2 Aboca SpA Società Agricola, Innovation & Medical Science Division, Loc. Aboca 20, 52037 Sansepolcro, Italy; LMattoli@aboca.it (L.M.); STamimi@aboca.it (S.T.); EFlamini@aboca.it (E.F.); SGaretto@naturalbiomedicine.it (S.G.); JLucci@naturalbiomedicine.it (J.L.); EGiovagnoni@aboca.it (E.G.); 3 Natural Bio-Medicine SpA, Loc. Aboca 20, 52037 Sansepolcro, Italy

**Keywords:** *Centella asiatica*, intra-articular treatment, osteoarthritis, MIA, RAW 264.7 cell line, 14G1862, pain

## Abstract

Current pharmacological therapies for the management of chronic articular diseases are far from being satisfactory, so new strategies need to be investigated. We tested the intra-articular pain relieving properties of a system of molecules from a characterized *Centella asiatica* extract (14G1862) in a rat model of osteoarthritis induced by monoiodoacetate (MIA). 14G1862 (0.2–2 mg mL^−1^) was intra-articularly (i.a.) injected 7 days after MIA, behavioural and histological evaluations were performed 14, 30 and 60 days after treatments. Moreover, the effect of 14G1862 on nitrate production and iNOS expression in RAW 264.7 macrophages stimulated with LPS was assessed. In vitro, 14G1862 treatment attenuated LPS-induced NO production and iNOS expression in a comparable manner to celecoxib. In vivo, 14G1862 significantly reduced mechanical allodynia and hyperalgesia, spontaneous pain and motor alterations starting on day 14 up to day 60. The efficacy was higher or comparable to that evoked by triamcinolone acetonide (100 μg i.a.) used as reference drug. Histological evaluation highlighted the improvement of several morphological parameters in MIA + 14G1862-treated animals with particularly benefic effects on joint space and fibrin deposition. In conclusion, i.a. treatment with *Centella asiatica* is a candidate to be a novel effective approach for osteoarthritis therapy.

## 1. Introduction

Osteoarthritis (OA) is a chronic degenerative joint disease typically affecting the knee, hips, fingers, and/or lower spinal regions [1] and is clinically characterized by gradual development of fluctuating joint pain, swelling, stiffness, and loss of motion [2]. Prevalence increases with age, making OA a leading cause of disability. Worldwide, the prevalence among individuals aged 60 years and older is estimated at 18% of women and 10% of men [3,4]. Approximately 80% of these individuals will have limitation of movements, and 1 in 4 will have impairments in major activities of daily living [3].

The options for OA treatment are generally classified as pharmacologic, nonpharmacologic, surgical, complementary and/or alternative [5]. Typically, patients are treated with a combination of these options in an attempt to achieve optimal results [6]. The pharmacological approach is usually based on symptom severity and duration, with the aim of relieving pain and slowing progression of the pathology [7,8,9]. Oral acetaminophen and nonsteroidal anti-inflammatory drugs (NSAIDs) are the mainstay for the treatment of OA. However, all of these agents have potential gastrointestinal, hepatic, and cardio-renal adverse effects [10] which increase with dosage and duration of treatment. Intra-articular treatment is another option approved by the Food and Drug Administration (FDA) for OA management that shows a number of advantages over systemic delivery, including increased local bioavailability, reduced systemic exposure, fewer adverse events and cost [11]. Intra-articular administration of immediate release (suspension) corticosteroids, including triamcinolone acetonide, has been routine in the management of knee OA; however, they cannot be administered more than 3 to 4 times per year due to side effects, and their efficacy remains controversial [12].

The necessity of novel interventions is warranted, and systems of molecules of natural origin can represent an effective source of therapeutic solutions [13]. So far, these systems have been infrequently used, given the fact that their potential has been poorly understood or unsatisfactorily explained. It appears evident that the exigency of a progressive evolution of the approach used was more rigorous and scientific.

*Centella asiatica*, known by the common name Gotu kola, is an important medicinal herb that is widely used in the orient and is becoming popular in the West. Apart from wound healing, the herb is utilized for the treatment of various skin conditions such as leprosy, lupus, varicose ulcers, eczema, psoriasis, diarrhoea, fever, amenorrhea, diseases of the female genitourinary tract and for improving cognition and relieving anxiety [14,15]. *Centella asiatica* has also been effective in chronic venous insufficiency by improvement of microcirculation [16]. Although various biological effects of *Centella asiatica* have been reported, its efficacy in relieving articular pain has not been investigated yet.

In this study we analysed, in vitro, the effect of a novel system of molecules called 14G1862 obtained from a characterized *Centella asiatica* extract. We tested 14G1862 effect on nitric oxide production, iNOS and COX-2 activation in macrophages RAW 264.7 cell line stimulated by LPS. Additionally, in an in vivo setup, we investigated the pain relief properties over two months of the same system of molecules after a single intra-articular injection in a rat model of osteoarthritis induced by MIA tibio-tarsal administration. Its efficacy was compared with the effect induced by a single i.a. injection of triamcinolone acetonide being the reference drug for the management of OA in the clinical setting. Moreover, we also performed the histological analysis of the joint at different time points from the beginning of the treatment in order to highlight a possible protective effect of the extract.

## 2. Materials and Methods

### 2.1. Preparation of the System of Molecules 14G1862 from Centella asiatica

The production process was performed in accordance with protocol described in the publicly available patent WO2018/138678A1 (https://patents.google.com/patent/WO2018138678A1/en?oq=WO2018%2f138678A1). Ethanol 96.4% from grain was of food grade. Purified water by means of an industrial Water Treatment Plants was produced from drinking water. Briefly, dried *Centella asiatica* leaves were subjected to extraction with ethanol 70% (ethanol:water 70:30 *v*/*v*) for 8 h at 50 °C and filtered to remove solid exhausted material. The resulting clarified extract was concentrated by ethanol evaporation under vacuum, until reaching the concentration factor of 7:1 (*v*:*v*, initial alcoholic extract volume compared to the volume after the evaporation step), then freeze-dried for 72 h. The resulting extract was stored until use, away from light and moisture.

### 2.2. Characterization Method of the System of Molecules 14G1862 from Centella asiatica

A comprehensive characterization of the resulting 14G1862 composition in terms of a broad spectrum of components such as different phenols, terpenes, aromatic alcohols, aldehydes, acids, esters and lactones, aromatic ketones, organic acids, fats, polysaccharides >20 KDa, vitamins and minerals was performed by using an advanced analytical platform principally based on mass spectrometry previously described [17]. Compounds identification was made using an in-house database of more than 1500 natural compounds. The most important polar compounds were processed by ultra-high-performance liquid chromatograph (UHPLC) coupled to a quadrupole time of flight (qToF) mass spectrometer. Volatile organic compounds (VOC) were characterized by means of gas chromatograph (GC) coupled to triple quadrupole (QqQ) mass spectrometer equipped with a Head Space sampler (HS) and a liquid split/splitless injector. Few fatty acids were characterized by means of gas chromatograph (GC) coupled to triple quadrupole (QqQ) mass spectrometer after derivatization with BSTFA reagent. Metals were characterized as elements by means of inductively coupled plasma (ICP) single quadrupole (Q) mass spectrometer. Anions were characterized by means of ionic chromatograph (IC) coupled to a conductivity detector (CD). Tannins were analyzed as class applying a spectrophotometric method reported in [18]. Polysaccharides were analyzed as class by means of size exclusion chromatography (SEC) method on high performance liquid chromatograph (HPLC) coupled to a refractive index detector (RID). All solvents were of high purity analytical grade and were used without further purification. ULC/MS grade absolute methanol was purchased from Biosolve (Dieuze, France). Ultrahigh purified water was prepared in a PURELAB^®^ Ultra water purification system (ELGA, UK). Formic acid 98%–100% for LC-MS LiChropur^®^ and dimethyl sulfoxide ≥99% were purchased from Sigma-Aldrich (St. Louis, MO, USA).

The high-purity reference standards, used both for the construction of the in-house database and for the construction of the calibration curves, were purchased from Extrasynthese (Genay, France), Sigma-Aldrich (St. Louis, MO, USA), PhytoLab GmbH & Co. KG (Vestenbergsgreuth, Germany) and ChromaDex (Irvine, CA, USA). High-purity reference standards stock solutions were prepared in methanol, water/methanol (80:20, *v*/*v*) or methanol/dimethyl sulfoxide (80:20, *v*/*v*) at 500 ppm. The working solutions were prepared by diluting appropriate volumes of the stock solutions with water/methanol (50:50, *v*/*v*). Internal standard sulfadimethoxine-*d*6 was purchased from Sigma-Aldrich (St. Louis, MO, USA). Internal standard stock solution was prepared in methanol at 5 ppm. All the stock solutions were stored in glass vials at −80 °C.

### 2.3. Cell Viability Assay

RAW 264.7 cells were seeded in 96-well plates at a density of 5 × 10^3^ cells/well in 100 μL of 10% FBS added medium. After 24 h incubation at 37 °C in 5% CO_2_, 14G1862 was added to the wells at a range of concentrations between 0.5–2 mg mL^−1^ to a final volume of 200 μL/well and incubated for 24 h at 37 °C in 5% CO_2_. Cell growth was assessed by the colorimetric method based on [3-(4,5-dimethylthiazol-2-yl)-5-(3-carboxymethoxyphenyl)-2-(4-sulfophenyl)-2H-tetrazolium, inner salt; MTS] and an electron coupling reagent (phenazine ethosulfate; PES) (Promega Corporation, WI, USA) The optical density of the chromogenic product was measured at 490 nm. Protein content was estimated using the Bio-Rad DC protein assay kit (Bio-rad, Segrate, Milan, Italy).

### 2.4. In Vitro Determination of Nitricoxide (NO) Production

The anti-inflammatory activity of 14G1862 extract was determined on the basis of NO production in macrophage culture supernatants using the Griess reaction [19]. RAW 264.7 cells (kindly provided by Prof. Bani, University of Florence and previously obtained from American Type Culture Collection (Rockville, MD, USA)) were cultivated in a 24-well microplate (1 × 10^6^ cells/well, in Dulbecco’s Modified Eagle Medium (DMEM; Euroclone, Milan, Italy) medium without phenol red containing 10% Fetal Bovine Serum (FBS; Euroclone, Milan, Italy). Cells were incubated at 37 °C in a 5% CO_2_ incubator until confluence. The cultured cells were treated with 14G1862 at the final concentration of 0.5, 1 and 2 mg mL^−1^ mixed with lipopolysaccharide solution (LPS, 1 μg mL^−1^). As positive control, well-known anti-inflammatory drug celecoxib (3 µM) was used. After incubating for 18 h at 37 °C, the cultured cell supernatant (100 μL) was mixed with an equal volume of Griess reagent (1% [*w*/*v*] sulfanilamide and 0.1% [*w*/*v*] N-[1-naphthyl] ethylenediamine hydrochloride in 2.5% [*v*/*v*] phosphoric acid) and incubated at RT for 10 min. The absorbance was measured at 540 nm using a microplate reader. NO production was calculated with reference to a standard curve obtained with NaNO_2_. Experiments were performed in triplicate.

### 2.5. RT-PCR

Total RNA from RAW 264.7 cells treated with described above was isolated using the miRNeasy Mini Kit (Qiagen Duesseldorf, Germany). For first-strand cDNA synthesis, 1μg of total RNA from each sample was reverse-transcribed. Primers were designed on the basis of the mouse GenBank sequences for cyclooxygenase-2 (COX2) [F 5′-TCT GCG ATG CTC TTC CGA GCT-3′; R 5′-GAT ACA CCT CTC CAC CGA TGA-3′ (751 BP)] and inducible NO synthase (iNOS) [F 5′-CGG ATA TCT CTT GCA AGT CCA AA-3′; R 5′-AAG TAT GTG TCT GCA GAT ATG-3′ (380 bp). Ribosomal protein large P1 (RPLP-1) was co-amplified as the reference [F 5′-ATCTACTCCGCCCTCATCCT-3′; R 5′-CAGATGAGGCTCCCAATGTT-3′ (155 bp)].

### 2.6. Animals

For all the experiments described below, male Sprague–Dawley rats (Envigo, Varese, Italy) weighing approximately 200–250 g at the beginning of the experimental procedure were used. Animals were housed in Ce.S.A.L. (Centro Stabulazione Animali da Laboratorio, University of Florence, Florence, Italy) and used at least one week after their arrival. Four rats were housed per cage (size 26 × 41 cm^2^); animals were fed a standard laboratory diet and tap water ad libitum, kept at 23 ± 1 °C with a 12 h light/dark cycle, light at 7 a.m. All animal manipulations were carried out according to the Directive 2010/63/EU of the European Parliament and of the European Union council (22 September 2010) on the protection of animals used for scientific purposes. The ethical policy of the University of Florence complies with the Guide for the Care and Use of Laboratory Animals of the US National Institutes of Health (NIH Publication No. 85-23, revised 1996; University of Florence assurance number: A5278-01). Formal approval to conduct the experiments described was obtained from the Italian Ministry of Health (No. 54/2014-B) and from the Animal Subjects Review Board of the University of Florence. Experiments involving animals have been reported according to ARRIVE guidelines [20]. All efforts were made to minimize animal suffering and to reduce the number of animals used.

### 2.7. MIA-Induced Osteoarthritis

Unilateral osteoarthritis was induced by injection of monoiodoacetate (MIA, Sigma-Aldrich, Milan, Italy) into the tibiotarsal joint [21,22]. On day 7, rats were slightly anesthetized by 2% isoflurane, the left leg skin was sterilized with 75% ethyl alcohol and the lateral malleolus located by palpation; then, a 28-gauge needle was inserted vertically to penetrate the skin and turned distally for insertion into the articular cavity at the gap between the tibio-fibular and tarsal bone until a distinct loss of resistance was felt. Two mg MIA in 25 μL saline was delivered into the left articular cavity. Control rats were treated with an equal volume of saline.

### 2.8. Treatment with the System of Molecules 14G1862 from Centella asiatica

14G1862 (0.2–2 mg mL^−1^) was dissolved in sterile saline solution and i.a. injected into the tibiotarsal joint 7 days after the articular damage induced by MIA. Briefly, rats were lightly anesthetized by 2% isoflurane, the left leg skin was sterilized with 75% ethyl alcohol, and the lateral malleolus located by palpation; then, a 28-gauge needle was inserted vertically to penetrate the skin and turned distally for insertion into the articular cavity at the gap between the tibiofibular and tarsal bone until a distinct loss of resistance was felt. A volume of 20 μL of solution was then injected (day 1) [23]. Control rats received 20 μL of saline solution. Behavioural measurements were performed on day 14, 30 and 60 after 14G1862 injection.

### 2.9. Paw Pressure Test

The nociceptive threshold in the rat was determined with an analgesimeter (Ugo Basile, Varese, Italy). Briefly, a constantly increasing pressure was applied to a small area of the dorsal surface of the hind paw using a blunt conical mechanical probe. Mechanical pressure was increased until vocalization or a withdrawal reflex occurred while rats were lightly restrained. Vocalization or withdrawal reflex thresholds were expressed in grams. These limits assured a more precise determination of mechanical withdrawal threshold in experiments aimed to ascertain the effect of treatments. An arbitrary cut-off value of 100 g was adopted. The data were collected by an observer who was blinded to the protocol [24,25].

### 2.10. Von Frey Test

The animals were placed in 20 × 20 cm plexiglas boxes equipped with a metallic meshy floor, 20 cm above the bench. A habituation of 15 min was allowed before the test. An electronic Von Frey hair unit (Ugo Basile, Varese, Italy) was used: the withdrawal threshold was evaluated by applying force ranging from 0 to 50 g with a 0.2 g accuracy. Punctuate stimulus was delivered to the mid-plantar area of each anterior paw from below the meshy floor through a plastic tip and the withdrawal threshold was automatically displayed on the screen. Paw sensitivity threshold was defined as the minimum pressure required to elicit a robust and immediate withdrawal reflex of the paw. Voluntary movements associated with locomotion were not taken as a withdrawal response. Stimuli were applied on each anterior paw with an interval of 5 s. The measure was repeated 5 times and the final value was obtained by averaging the 5 measures [26,27].

### 2.11. Incapacitance Test

Weight-bearing changes were measured using an incapacitance apparatus (Linton Instrumentation, Norfolk, UK) to detect changes in postural equilibrium after a hind limb injury [28,29]. Rats were trained to stand on their hind paws in a box with an inclined plane (65° from horizontal). This box was placed above the incapacitance apparatus, allowing us to independently measure the weight that the animal applied on each hind limb. The value reported for each animal is the mean of five consecutive measurements. In the absence of hind limb injury, rats applied equal weight on both hind limbs, indicating postural equilibrium, whereas an unequal distribution of weight on the hind limbs indicated a monolateral decreased pain threshold [21]. Data are expressed as the difference between the weight applied to the limb contralateral to the injury and the weight applied to the ipsilateral one (Δ weight).

### 2.12. Beam Balance Test

A balance beam test [30] consisted of the rats being placed on a narrowstrip of wood (30 cm × 1.3 cm) while balancing and the scoring standards were as follows: 0 point, the four limbs were all on the wood in a balance situations; 1 point, limbs of one side were able to grasp the wood or shake on the wood; 2 points, one or two limbs slipped from the wood; 3 points, three limbs slipped from the wood; 4 points, suspended on the wood and fell over after struggle [23].

### 2.13. Rota Rod Test

Rota-rod apparatus (Ugo Basile, Varese, Italy) consisted of a base platform and a rotating rod with a diameter of 6 cm and a non-slippery surface. The rod was placed at a height of 25 cm from the base. The rod, 36 cm in length, was divided into four equal sections by five disks. Thus, up to four rats were tested simultaneously on the apparatus, with a rod-rotating speed of 10 rpm. The integrity of motor coordination was assessed based on the number of falls from the rod for a maximum of 600 s. After a maximum of six falls from the rod, the test was suspended and the time was recorded. Each rat was assessed once, and the group mean average score was calculated [31].

### 2.14. Spontaneous Activity Meter (Animex Test)

Locomotor activity in rats was quantified using an Animex activity meter Type S (LKB, Farad, Sweden) set to maximum sensitivity. Every movement of rats, which were placed on the top of the Animex activity meter, produced a signal due to variation in inductance and capacity of the apparatus resonance circuit. Signals were converted automatically to numbers. On the day of the experiment, the cage, containing three rats, were put on the measuring platform. Activity counts were made for 5 min [21].

### 2.15. Histological Evaluations

Animals were killed by cervical dislocation. Legs were cut under the knee, flayed and fixed in 4% formaldehyde in phosphate-buffered saline (PBS) for 48 h at room temperature. Subsequently, samples were decalcified by 0.76 M sodium formate and 1.6 M formic acid solution in H_2_O for 4 weeks with a change of solution every 7 days. At the end of decalcification, these samples were routinely dehydrated in alcohol and embedded in paraffin. Sections (6 μM) thick were observed and an histological score (0: absent; 1: mild; 2: moderate; 3: severe) was attributed to the following morphological parameters: (a) inflammatory infiltrate; (b) synovial hyperplasia; (c) fibrin deposition; (d) synovial vascularity; (e) cartilage erosion; (f) bone erosion; (g) joint space [32,33].

### 2.16. Statistical Analysis

All the experimental procedures were performed by researchers blinded to the treatments. Each value represents the mean ± S.E.M of six rats per group, performed in two different experimental sets. The analysis of variance was performed by Analysis of Variance (ANOVA). A Bonferroni’s significant difference procedure was used as post hoc comparison. *p* values of less than 0.05 were considered significant. Data were analysed using the ‘Origin 9′ software (OriginLab, Northampton, MA, USA).

## 3. Results

### 3.1. Characterization of the System of Molecules 14G1862 from Centella asiatica

The results obtained showed how potent the analytical approach used to determine the molecular composition of 14G1862. Potassium, chloride, polysaccharides, madecassoside, asiaticoside, sodium, tannins and madecassic acids are the most abundant compounds found (Supplementary Figure S1).

Using different techniques principally based on mass spectrometry, ninety-three organic compounds, 24 elements and 4 anions were successfully identified from 14G1862, achieving a comprehensive chemical characterization of the system of molecules used in the experimental work (Table 1).

### 3.2. In Vitro Evaluation

**Figure 1:**
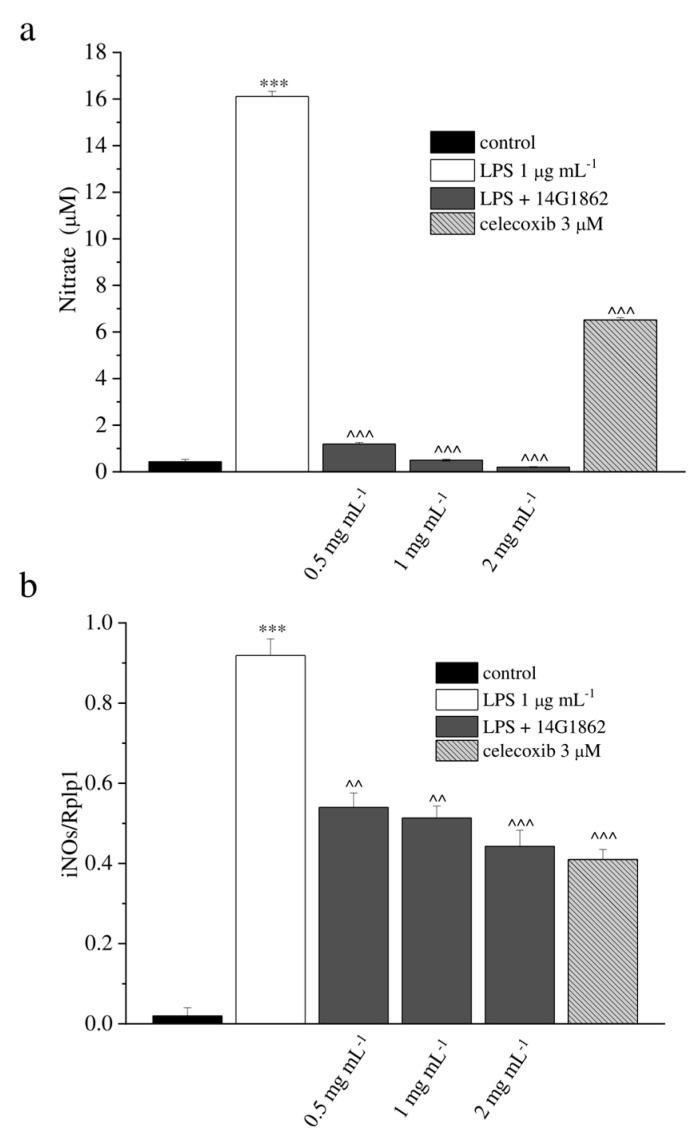
In-vitro antioxidant and anti-inflammatory properties of the system of molecules 14G1862 from *Centella asiatica*. (**a**) Effect of 14G1862 (0.5–2 mg mL^−1^), on nitrites production in RAW 264.7 macrophages stimulated with LPS 1 μg mL^−1^ for 18 h in comparison to celecoxib 3 μM. (**b**) Effect of 14G1862 (0.5–2 mg mL^−1^), on iNOS mRNA expression in RAW 264.7 macrophages stimulated with LPS 1 μg m^−1^ for 18 h in comparison to celecoxib 3 μM. Data are expressed as mean ± S.E.M. of three independent experiments. *** *p* < 0.001 vs. unstimulated control cells; ^ *p* < 0.001 and ^^^ *p* < 0.0001 vs. LPS-treated cells.

The effect of 14G1862 on NO production iNOS expression in RAW 264.7 macrophages stimulated with LPS is reported in Figure 1.

LPS significantly stimulated NO production by RAW 264.7 cells (*p* < 0.001). This effect was significantly decreased by 14G1862 in a concentration dependent manner (*p* < 0.001) as well as celecoxib 3 µM. (Figure 1a). In gene expression analysis, LPS significantly induce the expression of iNOS. 14G1862 significantly attenuated the LPS-induced expression of iNOS (*p* < 0.01 at 0.5 and 1 mg mL^−1^; *p* < 0.001 at 2 mg mL^−1^) (Figure 1b). None of the tested 14G1862 concentrations affected cell viability (Supplementary Table S1).

### 3.3. Behavioural Evaluation

**Figure 2:**
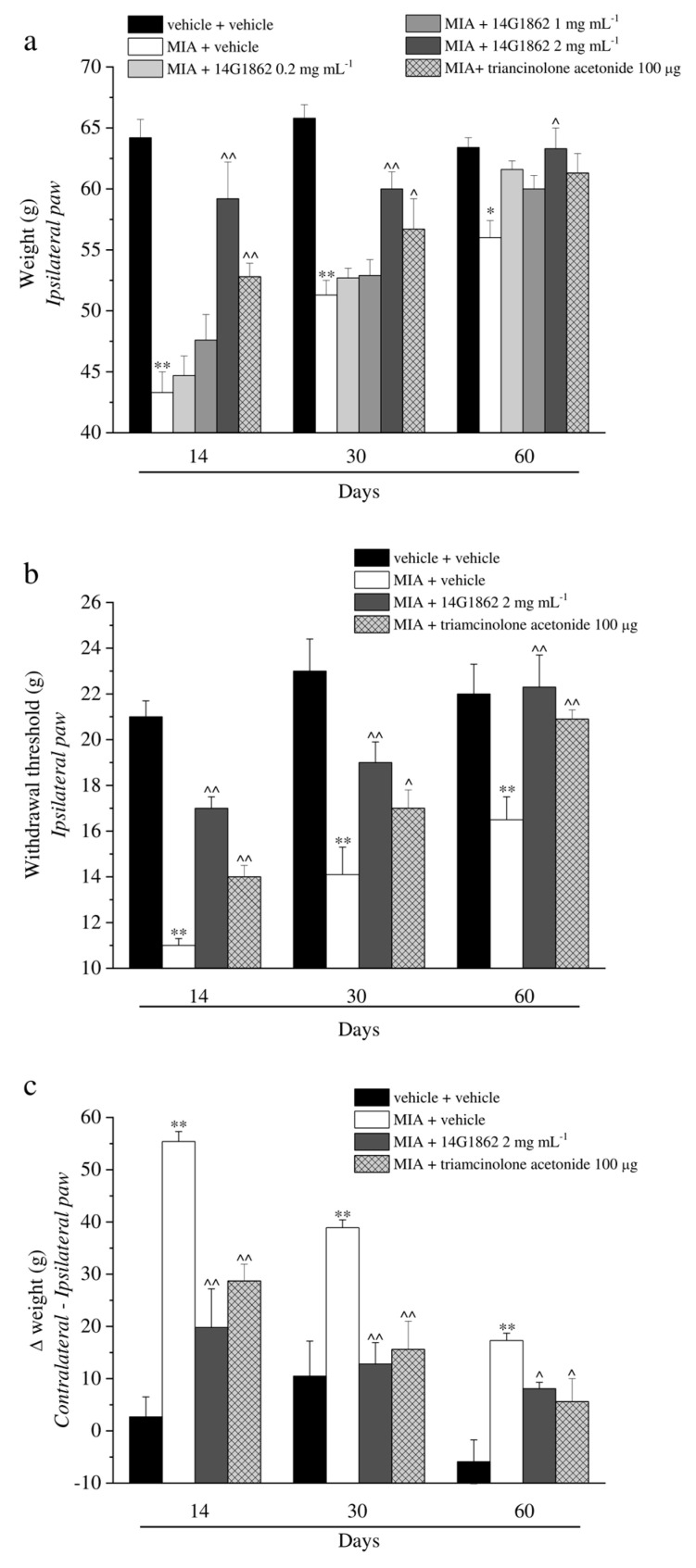
Articular pain, behavioural measurements related to hyperalgesia and allodynia. Monoarthritis was induced by MIA (2 mg/25 μL) injection into the tibio-tarsal joint on day 7. Twenty μL of 14G1862 (0.2–2 mg mL^−1^) or triamcinolone acetonide 100 μg were i.a. administered 7 days after MIA (day 1). Behavioural measurements were performed on days 14, 30 and 60 after osteoarthritis induction. The results obtained with the lower doses of 14G1862 were reported only in the Paw pressure test. (**a**) Effect of i.a. injection of 14G1862 in the rat, response to a noxious mechanical stimulus (Paw pressure test); (**b**) Effect of i.a. injection of 14G1862 in the rat, response to a non-noxious mechanical stimulus (Von Frey test); (**c**) Effect of i.a. injection of 14G1862 in the rat, measure of postural equilibrium related to pain (Incapacitance test). Each value represents the mean ± S.E.M. of 6 rats per group, performed in 2 different experimental sets. ** *p* < 0.01 vs. vehicle + vehicle; ^ *p* < 0.05 and ^^ *p* < 0.01 vs. MIA + vehicle.

The properties of a single i.a. injection of 14G1862 *Centella asiatica* extract was evaluated in the rat unilateral osteoarthritis model induced by MIA treatment. Behavioural measurements were performed on days 14, 30 and 60 from the damage. At these time points, the response to a mechanical noxious stimulus was measured by the Paw pressure test (Figure 2a). MIA injection significantly reduced the weight tolerated on the ipsilateral paw with respect to the vehicle + vehicle treated-animals on day 14 (43.3 ± 1.7 g vs. 64.2 ± 1.5 g, respectively). The hypersensitivity remained significantly lower in comparison to the control group on days 30 and 60 even if a progressive spontaneous recovery from pain was highlighted. The higher dose of 14G1862, once i.a. administered 7 days after MIA, significantly increased the withdrawal threshold of the ipsilateral paw up to about 60 g at all time points (Figure 2a). The lower dosages (0.2–1 mg mL^−1^) were ineffective. Triamcinolone acetonide was used as reference drug since its use in clinical to treat articular pain through i.a. injection. It is i.a. administration (100 μg) partially reduced the articular pain related by MIA injection on days 14 and 30 without reaching the value of the higher dose of 14G1862-treated animals. On day 60 it was ineffective. Figure 2b showed the response to a non-noxious mechanical stimulus evaluated by the Von Frey test. MIA injection decreased to 11.0 ± 0.3 g the withdrawal latency of the ipsilateral paw with respect to 21.0 ± 0.7 g of the control group on day 14, the animals showed a progressive spontaneous recovery, but the mechanical allodynia remained statistically significant until the end of the experiment. Only the effect of 2 mg mL^−1^ of 14G1862 was reported in the graph since the lower doses were inactive (Figure 2b). However, the results obtained with the lower doses in all tests are reported in the Supplementary Tables S2–S6. The extract partially rescued the animals from articular allodynia on days 14 and 30, and completely counteracted this condition on day 60. Triamcinolone acetonide was less effective but equally has reached the statistical significance. In both Paw pressure and Von Frey tests the pain sensitivity of the contralateral paw of MIA + vehicle or MIA+ treated groups was not different with respect to the control group (Supplementary Tables S7 and S8). Unilateral pain was also able to induce hind limb weight bearing alterations (Incapacitance test): the difference between the weight burdened on the contralateral paw and the ipsilateral one was significantly increased in MIA + vehicle group in comparison to control animals (vehicle + vehicle) from day 14 until day 60 (Figure 2c). 14G1862 counteracted the spontaneous pain measured as weight at all time points, showing a better profile with respect to triamcinolone treatment (Figure 2c).

**Figure 3:**
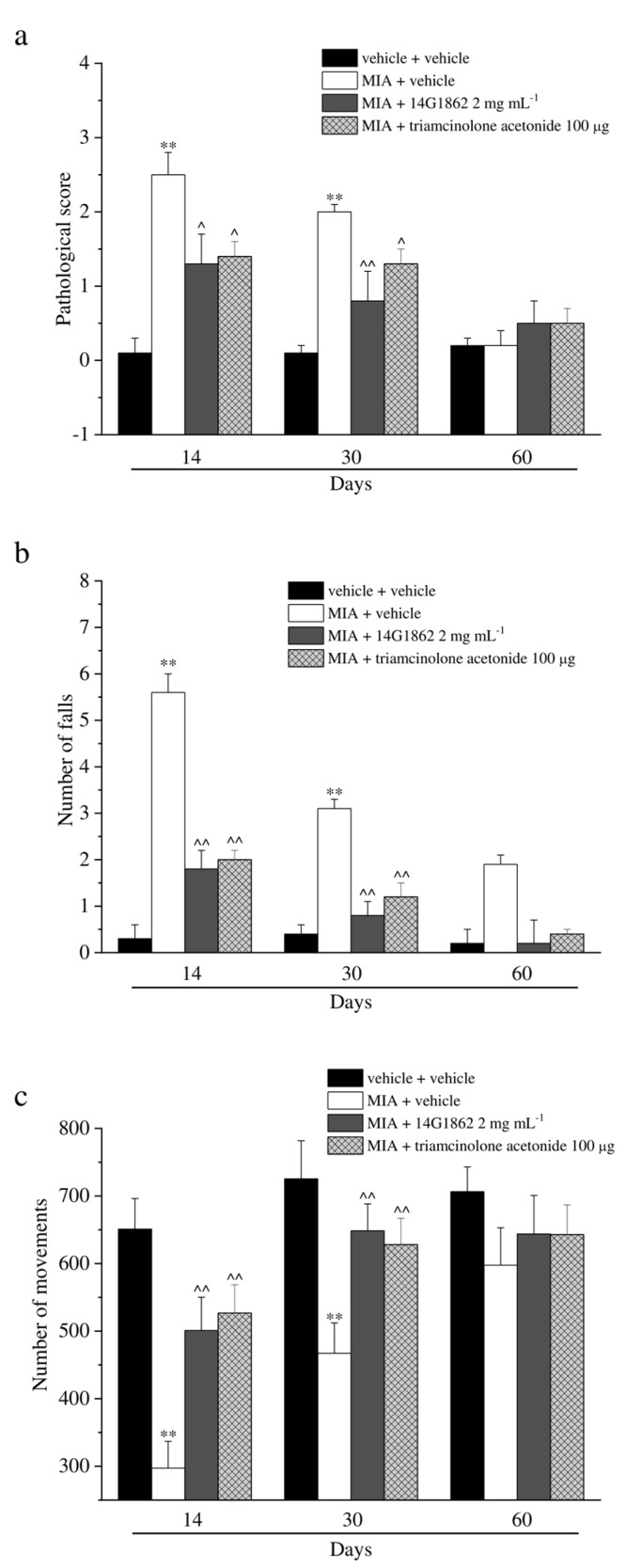
Articular pain, behavioural measurements related to motor alterations. Monoarthritis was induced by MIA (2 mg/25 μL) injection into the tibio-tarsal joint on day 7. Twenty μL of 14G1862 (2 mg mL^−1^) or triamcinolone acetonide 100 μg were i.a. administered 7 days after MIA (day 1). Behavioural measurements were performed on days 14, 30 and 60 after osteoarthritis induction. (**a**) Effect of i.a. injection of 14G1862 in the rat, measure of motor abilities related to pain (Beam balance test); (**b**) Effect of i.a. injection of 14G1862 in the rat, measure of number of falls in 10 min (Rota rod test); (**c**) Effect of i.a. injection of 14G1862 in the rat, measure of number of movements (Animex test). Each value represents the mean ± S.E.M. of 6 rats per group, performed in 2 different experimental sets. ** *p* < 0.01 vs. vehicle + vehicle; ^ *p* < 0.05 and ^^ *p* < 0.01 vs. MIA + vehicle.

MIA injection also evoked motor impairments and alterations as depicted in Figure 3. The score assigned to MIA + vehicle group by the Beam balance test was statistically increased with respect to the control animals on days 14 and 30, both treatments having significantly improved the motor skills, and reducing the score assigned to these groups. Similar results were obtained with the Rota rod and Animex tests (Figure 3b,c). Both tests highlighted a protective effect of 14G1862 treatment measured as a reduction of the number of falls of the animals and as an improvement of motor activity by the Rota rod test and Animex test, respectively. Also, triamcinolone acetonide treatment rescued the animals from motor impairments in a similar manner to 14G1862 (Figure 3a–c).

### 3.4. Histological Analysis

**Figure 4:**
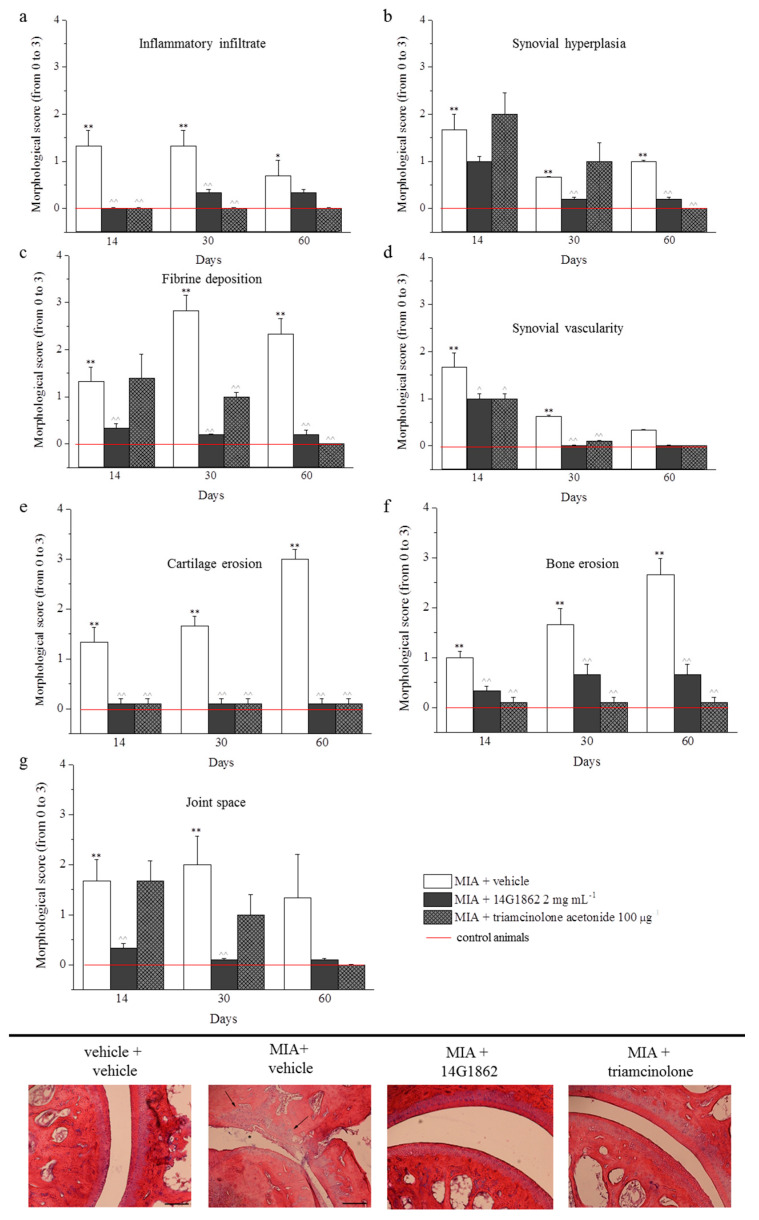
Morphological evaluations on tibio-tarsal joints. The upper panel (from **a** to **g**) represents the quantification of morphological parameters by specific score (0: absent; 1: light; 2: moderate; 3: severe). Control animals had all morphological score equal to 0 and were indicated in graphs by the red line. The lower panel shows comparative images of histological samples of tibio-tarsal joints from each experimental group. In the images are highlighted some morphological damages: cartilage and bone erosion by black arrows and fibrin deposition in joint space by asterisks. Magnification 100×; Scale bar 100 µM. Each value represents the mean ± S.E.M. of 6 rats per group, performed in 2 different experimental sets. ** *p* < 0.01 vs. vehicle + vehicle; ^^ *p* < 0.01 vs. MIA + vehicle.

The treatment with MIA was characterized by the presence of fibrin deposition in the joint space and several foci of cartilage and bone erosion. Moreover, the histological evaluation of synovia highlighted the presence of an abundant inflammatory infiltrate, a significant synovial hyperplasia and an increased synovial vascularity. The effect of 14G1862 on morphological derangement of tibio-tarsal joint were evaluated in repeatedly treated animals after 14, 30 and 60 days. Overall 14G1862 had an effect similar to that obtained by triamcinolone administration. It was in fact able to significantly reduce several morphological parameters such as the presence of inflammatory infiltrate, the synovial hyperplasia and vascularity, the bone and cartilage erosion (Figure 4). In two morphological parameters: joint space and fibrin deposition, the treatment with 14G1862 was more effective that one with triamcinolone. Animals treated with 14G1862, in fact, showed a lower fibrin deposition and a joint space preserved from the fourteenth day of treatment; on the contrary, triamcinolone acetonide ameliorated these parameters only 30 days after treatment (Figure 4).

## 4. Discussion

The present study shows the long lasting pain relieving properties of a single i.a. injection of a standardized 14G1862 *Centella asiatica* extract in a rat model of articular pain induced by MIA administration. Moreover, the histopathological analysis highlighted a protective effect of the treatment in reducing several morphological alterations of the joint at different time points after treatment (14, 30 and 60 days). The treatment efficacy is compared to that evoked by triamcinolone acetonide in the same animal pain model. Moreover, in vitro, the same system of molecules reduced NO production and iNOS expression in RAW 264.7 macrophages cell line stimulated with LPS.

OA is a heterogeneous condition characterized by a complex and multifactorial etiology which contributes to the broad clinical variations in symptoms presentation and treatment responses [34]. This postures a challenge for the identification of effective treatment for OA. So far, there have been no effective treatments in withdrawing OA disease progression and modifying structural development at advanced disease stages. Pharmacological treatments are mainly palliative targeting pain reduction in early stage of OA. The most common drugs used are acetaminophen, non-steroidal anti-inflammatory drugs, tramadol, intra-articular injection of corticosteroid and hyaluronic acid, i.e., viscosupplementation [35,36]. The American College of Rheumatology (ACR) and the Osteoarthritis Research Society International (OARSI) [9,37] recommend intra-articular corticosteroids for short-term management of OA pain, although evidence of improvement in pain, stiffness, and disability versus placebo has been controversial [38]. Adverse reactions to corticosteroid injections exist; Chandler and Wright first described radiographic evidence of destruction of the knee joint and cartilage after several corticosteroid injections [39]. The incidence of joint infection following corticosteroid administration is rare but may be as high as one in three thousand patients, with an associated mortality rate of approximately 11%. Additional known complications include pain, skin atrophy, tendinopathy and systemic hyperglycemia [40].

In the last few years, there has been an exponential growth of interest in systems of molecules with therapeutic effect, gaining popularity in both developing and developed countries, because of their natural origin, reduced risk of side effects, effectiveness with chronic conditions, lower cost and widespread availability. Medicinal plants, in fact, contain dozens of systems with different therapeutic effects that are able to offer a multiple approach to the complexity of the disease. In the present research, a single i.a. injection of 14G1862 reduced articular pain induced by MIA injection up to 60 days after treatment in a dose dependent manner. It was able to reduce mechanical hyperalgesia and allodynia, spontaneous pain, and, finally, to counteract motor impairments typical of monolateral articular pain. The therapeutic effect reached by the single i.a. treatment with the extract was compared to that obtained by triamcinolone acetonide (100 μg). Results highlighted a comparable effect achieved between the two groups and in tests like Paw pressure and Von Frey the system of molecules appears to be even more effective during the first two weeks. The articular pain model used is characterized by a first inflammatory phase (day 7 after MIA injection) followed by an increasing neuropathic component leading to enhanced response to normal or suprathreshold stimulation (allodynia and hyperalgesia measurements respectively) [21]. Moreover, the intra-articular injection of MIA induces necrosis of chondrocytes with a decrease of cartilage thickness and osteolysis [41], in the presence of a considerable component of oxidative stress [42]. Kobayashi et al. [43] showed that MIA is able to disorganize chondrocytes and to promote cartilage erosion [44,45]. These alterations are comparable with joint damages typical of humans affected by osteoarthritis and very consistent with those recorded in the presently performed histological analysis. 14G1862 relieved MIA-dependent articular pain both during the early inflammatory than the neuropathic pain states and it was able to rescue the joint from several morphological alterations such as the presence of inflammatory infiltrate, the synovial hyperplasia and vascularity, the bone and cartilage erosion. Moreover, as regards two morphological parameters (joint space and fibrin deposition), treatment with 14G1862 was more effective that one with triamcinolone. Indeed, the animals treated with the system of molecules showed a lower fibrin deposition and a joint space preserved from the fourteenth day of treatment; on the contrary, triamcinolone ameliorated these parameters only 30 days after treatment. Several pathways are involved in the pathogenesis of OA. Mechanical damage can cause a localized inflammatory response of the joint, marked by increased of pro-inflammatory mediators such as interleukin-1β (IL-1β), interleukin-6 (IL-6), tumor necrosis factor-α (TNF-α), nitrite oxide (NO) and prostaglandin E2 (PGE2) in the joint space [46,47,48,49,50]. This inflammatory response further exaggerates cartilage tissue damage via oxidative stress and damage, thus forming a vicious self-destructive cycle. Oxidative stress is also related to OA, as evidenced by an upregulation of inducible NO synthase (iNOS) and nicotinamide adenine dinucleotide phosphate oxidase in chondrocytes [51]. These enzymes produce high levels of reactive oxygen and nitrogen species (ROS and RNS), including NO, superoxide anion, peroxynitrite and hydrogen peroxide (H_2_O_2_) [52,53,54,55,56,57]. The cellular antioxidant enzymes have been found to be compromised in animal models and patients with OA [58,59,60,61]. An imbalance between oxidants and antioxidants results in oxidative damage, endoplasmic reticulum stress and mitochondrial dysfunction in chondrocytes (intrinsic pathway of apoptosis) [62,63,64], which subsequently leads to chondrocytic differentiation or apoptosis. These data suggest that the complexity of the mechanisms responsible for OA development requires multitarget treatments. In our in-vitro studies, 14G1862 significantly reduced LPS-induced NO production in RAW 264.7 macrophages cell line at all doses tested highlighted antioxidant/anti-inflammatory properties. LPS treatment is commonly used in order to evoke an inflammatory reaction in macrophages simulating a bacterial infection. Moreover, LPS is a useful model to test the anti-inflammatory effects of several compounds [19]. This result matched with that obtained upon LPS stimulation for 18 h by RT-PCR in which the extract was able to counteract the induction of iNOS expression in the same cells. Data obtained from in-vitro evaluations highlighted a possible biphasic effect of 14G1862. In fact, the extract reduced the amount of nitrite in the culture media acting both as antioxidant (NO scavenging) and as anti-inflammatory agent (iNOS expression modulating). This dual effect might be explained by the complex composition of the system. For example, literature data showed that flavonoids may act as NO scavenger [65], and phenols may play a role in iNOS expression modulation [66]. The extract used was characterized by the presence of phenols (2.6%), of which flavonoids, phenylpropanoid derivatives and salicylates are the main constituents, terpenes (5.0%), polysaccharides (5.7%) and minerals (16.5%). The antioxidant properties highlighted could be related to the presence of flavonoids as quercetin-3-O-glucoronide, quercetin-3-O-glucopyranoside and kaempferol [67,68]. Borghi and colleagues [69] studied the effects of intraperitoneal treatment of quercetin (10–100 mg/kg) in a rat model of TiO_2_-induced arthritis. The flavonoid was able to inhibit in a dose-dependent manner TiO_2_-induced knee joint mechanical hyperalgesia, leukocyte recruitment and edema. Histopathological changes such as leukocyte infiltration, vascular proliferation and synovial hyperplasia (pannus formation) on day 30 after TiO_2_ challenge were recorded and the protective analgesic and anti-inflammatory mechanisms of quercetin included the activation of Nrf2/HO-1 signaling pathway, as well as the inhibition of TiO_2_-induced neutrophil and macrophage recruitment, proteoglycan degradation, oxidative stress, cytokine production (TNF-α, IL-1β, IL-6, and IL-10), COX-2 mRNA expression, and bone resorption [69]. In another study, the presence of flavonoids in a *Polygonum orientale* extract were considered relevant in exerting anti-inflammatory and analgesic effects in a rat model of articular pain induced by Complete Freund’s Adjuvant and carrageenan, respectively [70]. However, other mechanisms besides antioxidants cannot be excluded. As reported by the literature, the presence of phenylpropanoids and their derivatives can exert multifaceted effects which include being anti-inflammatory [71]. Nevertheless, also triterpenes are implicated in reducing OA-induced pain and joint destruction. As shown by Kao and colleagues, a shea nut oil enriched with triterpenes was capable of relieving the symptoms of OA and protecting the cartilage from degeneration in rats, suggesting a future nutraceutical application to prevent articular pain and damages [72]. A recent study highlighted the efficacy and the mechanism of action of polygalacid, a triterpene obtained from the root of *Polygala tenuifolia* Wild, in in-vitro and in-vivo studies. The triterpene reduced the expression of MMPs and COX-2 in rat chondrocytes suppressing the activation of Wnt/β-catenin and the mitogen-activated protein kinase (MAPK) signal pathway [73].

Yet, a system of molecules of natural origin displays an intrinsic complexity capable of leading to the impossibility to define the molecular underpinnings underlying its efficacy by applying the conceptual tools typical of the study single, isolated molecules exerting pharmacological effects, such as the key-lock model. Novel epistemological tools, capable of accommodating for this intrinsic complexity at both the structural and functional level, will have to be developed [74].

## 5. Conclusions

In conclusion, a single i.a. injection of the system of molecules 14G1862 from *Centella asiatica* extract was able to counteract mechanical hyperalgesia, allodynia and motor alterations in a rat model of MIA-induced osteoarthritis. The preparation also reduced the NO unbalance in macrophages cell line stimulated by LPS. The system of molecules 14G1862 from *Centella asiatica* by i.a. route is therefore suggested as a new candidate to be further validated for the treatment of articular pain.

## 6. Patents

WO2018/138678A1.

## Figures and Tables

**Table 1:** Composition study of the system of molecules 14G1862 from Centella asiatica.

Methods	Compounds	% *(g of Compound/100 g of Sample)*
	**Phenols, Total**	**2.3409**
	***Of which Flavonoids, total***	**0.430**
	*Of which Flavonols, total*	**0.2910**
UHPLC q-ToF	Kaempferol-7-O-glucoside	0.019
UHPLC q-ToF	Quercetin-3-O-glucuronide	0.18
UHPLC q-ToF	Quercetin-3-O-glucopyranoside	0.033
UHPLC q-ToF	Rutin	0.02
UHPLC q-ToF	Isorhamnetin	0.0390
	*Of which Flavanons, total*	**0.0100**
UHPLC q-ToF	Flavanomarein	0.01
	*Of which Dihydrochalcones, total*	**0.0140**
UHPLC q-ToF	Phloridzin	0.014
	*Of which Flavones, total*	**0.1150**
UHPLC q-ToF	Luteolin-4’-O-glucoside	0.091
UHPLC q-ToF	Luteolin-7-O-β-D-glucoside	0.012
UHPLC q-ToF	Schaftoside	0.012
	***Of which Acid Phenol, total***	**0.0230**
UHPLC q-ToF	Protocatechuic acid	0.023
	***Of which Phenylpropanoid derivatives, total***	**0.48344**
	*Of which Hydroxycinnamic acids, total*	**0.0228**
GC-MS (HS)	Cinnamic acid ethyl ester	0.00006212
GC-MS (HS)	Cinnamic acid methyl ester	0.00004905
UHPLC q-ToF	Verbascoside	0.0130
UHPLC q-ToF	Rosmarinic acid	0.0097
	*Of which Monolignols, total*	**0.0068**
UHPLC q-ToF (pos)	Syringin (Eleutheroside B)	0.0068
	*Of which Coumarins, total*	**0.1110**
UHPLC q-ToF	Aesculetin	0.064
UHPLC q-ToF	Aesculin	0.034
UHPLC q-ToF	Fraxin	0.013
	*Of which Lignans and Phenylpropenes, total*	**0.000770045**
GC-QqQ (HS)	Acetyleugenol	0.00011256
GC-QqQ (HS)	β-Asarone	0.00011564
GC-QqQ (HS)	α-Asarone	0.00006897
GC-QqQ (HS)	Apiole	0.00006139
GC-QqQ (HS)	Dillapiole	0.00006708
GC-QqQ (HS)	Eugenol	0.00017197
GC-QqQ (HS)	Isoeugenyl acetate	0.00013133
GC-QqQ (HS)	Myristicin	0.00004111
	*Of which Caffeic acids derivatives, total*	**0.342**
UHPLC q-ToF	3,4-Dicaffeoylquinic acid	0.022
UHPLC q-ToF	4,5-Dicaffeoylquinic acid	0.13
UHPLC q-ToF	Chlorogenic Acid	0.190
	***Of which Salicylates, total***	**0.027**
UHPLC q-ToF	Salicylic acid	0.027
	***Of which Simple Phenols, total***	**0.0000441**
GC-QqQ (HS)	*m*-Cresyl Acetate	0.00001636
GC-QqQ (HS)	Guaiacol Methyl ether	0.00002774
	***Of which Xanthones, total***	**0.0075**
UHPLC q-ToF	Mangiferin	0.0075
SFM	***Tannins, total***	***1.37***
	**Terpenes, Total**	**5.012665**
	***Of which Monoterpenes*, total**	**0.004806**
	*Of which Monoterpene simple, total*	**0.001123**
GC-QqQ (HS)	β-Curcumene	0.00068904
GC-QqQ (HS)	*p*-Cymene (4-Cymene)	0.00017318
GC-QqQ (HS)	α-Curcumene	0.00007149
GC-QqQ (HS)	Limonene	0.00004334
GC-QqQ (HS)	β-Myrcene (myrcene)	0.00007774
GC-QqQ (HS)	β-Pinene	0.00003455
GC-QqQ (HS)	ɣ-Terpinene	0.00003338
	*Of which Monoterpene alcohol, total*	**0.002889101**
GC-QqQ (HS)	trans-Anethole	0.00004189
GC-QqQ (HS)	Borneol	0.00076609
GC-QqQ (HS)	1,8-Cineol (Eucalyptol)	0.00002888
GC-QqQ (HS)	Geranyl Acetate	0.00037654
GC-QqQ (HS)	Linalool	0.00143459
GC-QqQ (HS)	Terpinen-4-ol	0.00020283
GC-QqQ (HS)	Menthol	0.00003828
	*Of which Monoterpene ketones and aldehydes total*	**0.00056306**
GC-QqQ (HS)	Camphor	0.00027296
GC-QqQ (HS)	Carvone	0.00029010
	*Of which Monoterpene Phenols derivatives, total*	**0.00023069**
GC-QqQ (HS)	Carvacrol	0.00012183
GC-QqQ (HS)	Thymol	0.00010886
	***Of which Sesquiterpenes*, total**	**0.00050243**
GC-QqQ (HS)	Guaiazulene	0.00000929
GC-QqQ (HS)	Valencene	0.00000851
GC-QqQ (HS)	β-Eudesmol	0.00002983
GC-QqQ (HS)	Cedrol	0.00002645
GC-QqQ (HS)	Guaiol	0.00003122
GC-QqQ (HS)	Alloaromadendrene	0.00021006
GC-QqQ (HS)	α-Humulene	0.00011879
GC-QqQ (HS)	α-Bisabolol	0.00003985
GC-QqQ (HS)	Azulene	0.00001726
GC-QqQ (HS)	Chamazulene	0.00001117
	***Of which Triterpenes*, *total***	**5.00724**
UHPLC q-ToF	Asiatic Acid	0.087
UHPLC q-ToF	Madecassic Acid	0.660
	*Of whichSaponins, total*	**4.2602**
UHPLC q-ToF	Asiaticoside	1.96
UHPLC q-ToF	Madecassoside	2.30
UHPLC q-ToF	Hederagenin	0.00024
	***Of which Apocarotenoids*, *total***	**0.00011726**
GC-QqQ (HS)	β-Ionone	0.00008004
GC-QqQ (HS)	α-Ionone	0.00003722
	**Aromatic Alcohols, Total**	**0.00017948**
GC-QqQ (HS)	1-Phenylethanol	0.00003749
GC-QqQ (HS)	4-Isopropyl Benzyl Alcohol	0.00014199
	**Aromatic Aldehydes, Total**	**0.0003053**
GC-QqQ (HS)	Anisaldehyde	0.00026692
GC-QqQ (HS)	Cuminaldehyde	0.00003838
	**Aromatic Acids, Aromatic Esters and Lactones, Total**	**0.00023445**
GC-QqQ (HS)	Phenylacetic Acid Ethyl ester	0.00003081
GC-QqQ (HS)	Benzoic acid Ethyl ester	0.00002015
GC-QqQ (HS)	Benzyl Acetate	0.00002002
GC-QqQ (HS)	Benzyl Benzoate	0.00001766
GC-QqQ (HS)	Benzoic acid Eugenyl Ester	0.00001478
GC-QqQ (HS)	Benzoic Acid Methyl ester	0.00000798
GC-QqQ (HS)	Cinnamyl Acetate	0.00012305
	**Aromatic Ketones Total**	**0.00009413**
GC-QqQ (HS)	4-Chromanone	0.0000718
GC-QqQ (HS)	Acetophenone	0.0000223
	**Esters, Total**	**0.00003215**
GC-QqQ (HS)	Ethyl Decanoate	0.00003215
	**Organic Acids, Total**	**0.16**
	***Of which Monocarboxylic*, *total***	**0.13**
UHPLC q-ToF	Quinic acid	0.13
	***Of which Dicarboxylic*, *total***	**0.032**
UHPLC q-ToF	Azelaic acid	0.032
	**Fats, Total**	**0.002828**
	***Of which Saturated acids and derivatives,* total**	**0.0093**
GC-QqQ (Der)	Lignoceric Acid	0.00220
GC-QqQ (Der)	Myristic Acid	0.00160
GC-QqQ (Der)	Behenic Acid	0.00300
GC-QqQ (Der)	Arachidic Acid	0.00240
GC-QqQ (Der)	Octanoic Acid	0.00009
	***Of which Unsaturated acids and derivatives,* total**	**0.018990**
GC-QqQ (Der)	Oleic Acid	0.00089
GC-QqQ (Der)	Linoleic Acid	0.01810
HPLC-RID	**Polysaccharides > 20KDa, *Total***	**5.74**
	**Vitamins, *Total***	**0.35**
	***Of which Hydro-soluble Vitamins***	**0.35**
UHPLC q-ToF (pos)	Choline (Vit J)	0.35
	**Minerals, *Total***	**16.518931**
	***Of which Macro-elements, Total***	**8.9494**
ICP-MS	Calcium	0.0614
ICP-MS	Magnesium	0.2183
ICP-MS	Potassium	7.0843
ICP-MS	Sodium	1.5854
	***Of which Micro- Oligo-elements, Total***	**0.02184**
ICP-MS	Cobalt	0.000172
ICP-MS	Copper	0.000757
ICP-MS	Iron	0.0028
ICP-MS	Manganese	0.0053
ICP-MS	Selenium	0.000001
ICP-MS	Zinc	0.001016
ICP-MS	Arsenic	0.000006
ICP-MS	Boron	0.000439
ICP-MS	Chromium	0.000027
ICP-MS	Nichel	0.000608
ICP-MS	Silicon	0.0107
ICP-MS	Vanadium	0.0000140
	***Of which Other elements, Total***	**0.0293**
ICP-MS	Rubidium	0.02217
ICP-MS	Lithium	0.000063
ICP-MS	Barium	0.000103
ICP-MS	Aluminium	0.0069
ICP-MS	Cadmium	0.0000190
ICP-MS	Thallium	0.0000260
ICP-MS	Gallium	0.0000090
ICP-MS	Selenium	0.0000010
	***Of which Anions, Total***	**7.1584**
IC-CD	Chloride	6.8162
IC-CD	Nitrate	0.3251
IC-CD	Phosphate	0.2086
IC-CD	Sulphate	0.1685

## Supplementary Materials

The following are available online at https://www.mdpi.com/2072-6643/12/6/1618/s1, Figure S1: System of molecules 14G1862, freeze-dried extract UHPLC-qToF fingerprint Total Ion Current_ Negative ions. b. System of molecules 14G1862 freeze-dried extract UHPLC-qToF fingerprint_Total Ion Current_ Positive ions. c. System of molecules 14G1862 freeze-dried extract GC-QqQ (HS) fingerprint Total ion Current_ MRM Mode. d. System of molecules 14G1862 freeze-dried extract ICP-MS fingerprint _Total Ion Current. e. System of molecules 14G1862 freeze-dried extractIC-CD_Anions fingerprint.
