# Supplementary material for: Intra-Articular Route for the System of Molecules 14G1862 from Centella asiatica: Pain Relieving and Protective Effects in a Rat Model of Osteoarthritis
Source: Nutrients. 2020 May 31;12(6):1618. doi: 10.3390/nu12061618 (PMC7352185; doi:10.3390/nu12061618)
Supplement: Supplementary file 1 [file nutrients-12-01618-s001.pdf]

Supplementary Table S1. Cell viability assay

	Cell Viability %
14G1862	24 h Incubation
0	100 ± 0.3
0.5 mg mL^{-1}	103 ± 0.4
1 mg mL^{-1}	102 ± 1.9
2 mg mL^{-1}	98 ± 1.4

RAW 264.7 cells were treated with increasing concentration of 14G1862 for 24 h. cell viability was measured by the MTS assay. The control condition was arbitrarily set as 100% and value are expressed as the mean ± S.E.M. of three independent experiments.

	Withdrawal threshold (g), ipsilateral paw		
Treatments	Day 14	Day 30	Day 60
vehicle + vehicle	21.0 ± 0.7	23.6 ± 1.4	22.5 ± 1.3
MIA + vehicle	11.0 ± 0.3**	14.1 ± 1.2**	16.5 ± 1.0**
MIA + 14G1862 0.2 mg mL^{-1}	11.9 ± 0.8	16.4 ± 1.5	19.6 ± 2.1
MIA + 14G1862 1 mg mL^{-1}	12.6 ± 1.6	16.7 ± 0.6	18.4 ± 1.9

Supplementary Table S2. Von Frey test

Articular pain, response to a non noxious mechanical stimulus (Von Frey test). Twenty µl of 14G1862 (0.2 – 1 mg mL^{-1}) were i.a. administered 7 days after MIA (day 1). Behavioural measurements were performed on days 14, 30 and 60 after osteoarthritis induction. Each value represent the mean ± S.E.M of 6 rats per group, performed in 2 different experimental sets. **P<0.01 *vs* vehicle + vehicle

Supplementary Table S3. Incapacitance test

	Δ Weight (g) (contralateral paw – ipsilateral paw)		
Treatments	Day 14	Day 30	Day 60
vehicle + vehicle	0.3 ± 3.8	10.5 ± 6.7	-5.9 ± 4.2
MIA + vehicle	55.4 ± 1.9**	38.9 ± 1.5**	17.3 ± 1.4**
MIA + 14G1862 0.2 mg mL^{-1}	48.6 ± 9.4	33.6 ± 1.9	14.8 ± 2.4
MIA + 14G1862 1 mg mL^{-1}	39.3 ± 9.8	35.0 ± 5.6	12.3 ± 3.5

Articular pain, measure of postural equilibrium related to pain (Incapacitance test). Twenty µl of 14G1862 (0.2 – 1 mg mL^{-1}) or triamcinolone acetonide 100 µg were i.a. administered 7 days after MIA (day 1). Behavioural measurements were performed on days 14, 30 and 60 after osteoarthritis induction. Each value represent the mean ± S.E.M of 6 rats per group, performed in 2 different experimental sets. **P<0.01 *vs* vehicle + vehicle

Supplementary Table S4. Beam balance test

	Pathological score		
Treatments	Day 14	Day 30	Day 60
vehicle +	0.2 ± 0.1	0.1 ± 0.2	0.2 ± 0.2

vehicle			
MIA + vehicle	2.5 ± 0.3**	1.5 ± 0.4**	0.2 ± 0.2
MIA + 14G1862 0.2 mg mL^{-1}	2.7 ± 0.3	0.8 ± 0.7	0.5 ± 0.3
MIA + 14G1862 1 mg mL^{-1}	2.5 ± 0.3	1.0 ± 0.5	0.2 ± 0.2

Articular pain, measure of motor abilities related to pain (Beam balance test). Twenty µl of 14G1862 (0.2 – 1 mg mL^{-1}) were i.a. administered 7 days after MIA (day 1). Behavioural measurements were performed on days 14, 30 and 60 after osteoarthritis induction. Each value represent the mean ± S.E.M of 6 rats per group, performed in 2 different experimental sets. **P<0.01 *vs* vehicle + vehicle

Supplementary Table S5. Rota rod test

	Number of falls		
Treatments	Day 14	Day 30	Day 60
vehicle + vehicle	0.3 ± 0.3	0.4 ± 0.2	0.2 ± 0.3
MIA + vehicle	5.6 ± 0.4**	3.1 ± 0.2**	1.9 ± 0.2*
MIA + 14G1862 0.2 mg mL^{-1}	4.9 ± 0.5	2.7 ± 0.3	1.5 ± 0.5
MIA + 14G1862 1 mg mL^{-1}	4.4 ± 0.7	3.1 ± 0.2	0.9 ± 0.8

Articular pain, measure of motor coordination related to pain (Rota rod test). Twenty µl of 14G1862 (0.2 – 1 mg mL^{-1}) were i.a. administered 7 days after MIA (day 1). Behavioural measurements were performed on days 14, 30 and 60 after osteoarthritis induction. Each value represent the mean ± S.E.M of 6 rats per group, performed in 2 different experimental sets. **P<0.01 *vs* vehicle + vehicle

Supplementary Table S6. Animex test

	Number of movements		
Treatments	Day 14	Day 30	Day 60
vehicle + vehicle	651.0 ± 45.2	725.4 ± 56.4	706.4 ± 36.7
MIA + vehicle	297.3 ± 39.7**	467.2 ± 44.9**	597.6 ± 55.5
MIA + 14G1862 0.2 mg mL^{-1}	298.6 ± 50.8	529.1 ± 54.0	597.6 ± 51.0
MIA + 14G1862 1 mg mL^{-1}	326.4 ± 52.3	499.7 ± 59.1	668.9 ± 43.5

Articular pain, measure of spontaneous activity related to pain (Animex test). Twenty µl of 14G1862 (0.2 – 1 mg mL^{-1}) were i.a. administered 7 days after MIA (day 1). Behavioural measurements were performed on days 14, 30 and 60 after osteoarthritis induction. Each value represent the mean ± S.E.M of 6 rats per group, performed in 2 different experimental sets. **P<0.01 *vs* vehicle + vehicle

Supplementary Table S7. Paw pressure test

	Weight (g), contralateral paw		
Treatments	Day 14	Day 30	Day 60
vehicle + vehicle	62.1 ± 0.9	62.7 ± 1.2	63.7 ± 1.5
MIA +	65.0 ± 0.3	62.5 ± 1.4	65.8 ± 0.8

vehicle			
MIA + 14G1862 0.2 mg mL^{-1}	66.1 ± 2.3	61.6 ± 1.4	64.3 ± 1.5
MIA + 14G1862 1 mg mL^{-1}	62.9 ± 0.9	64.8 ± 2.3	61.9 ± 2.5
MIA + 14G1862 2 mg mL^{-1}	64.2 ± 0.8	63.3 ± 0.8	64.2 ± 0.8
MIA + triamcinolone acetonide 100 µg	66.1 ± 2.3	61.6 ± 1.4	64.3 ± 1.5

Evaluation of mechanical hyperalgesia on the contralateral paw by the Paw pressure test. Behavioural measurements were performed on days 14, 30 and 60 after osteoarthritis induction. Each value represent the mean ± S.E.M of 6 rats per group, performed in 2 different experimental sets.

Supplementary Table S8. Von Frey test

	Withdrawal threshold (g), contralateral paw		
Treatments	Day 14	Day 30	Day 60
vehicle + vehicle	22.5 ± 1.4	23.7 ± 2.8	24.6 ± 2.1
MIA + vehicle	22.7 ± 0.4	23.8 ± 0.8	23.4 ± 1.7
MIA + 14G1862 0.2 mg mL^{-1}	23.7 ± 1.6	23.7 ± 1.6	22.6 ± 1.3
MIA + 14G1862 1 mg mL^{-1}	20.8 ± 0.9	20.8 ± 0.9	21.8 ± 0.9
MIA + 14G1862 2 mg mL^{-1}	22.8 ± 0.6	25.1 ± 0.8	24.7 ± 1.7
MIA + triamcinolone acetonide 100 µg	23.4 ± 0.3	24.1 ± 0.3	24.5 ± 0.5

Evaluation of mechanical allodynia on the contralateral paw by the Von frey test. Behavioural measurements were performed on days 14, 30 and 60 after osteoarthritis induction. Each value represent the mean ± S.E.M of 6 rats per group, performed in 2 different experimental sets.
